# Conditional Responses of Benthic Communities to Interference from an Intertidal Bivalve

**DOI:** 10.1371/journal.pone.0065861

**Published:** 2013-06-18

**Authors:** Carl Van Colen, Simon F. Thrush, Magda Vincx, Tom Ysebaert

**Affiliations:** 1 Ghent University, Department of Biology, Marine Biology Research Group, Ghent, Belgium; 2 National Institute of Water and Atmospheric Research, Hamilton, New Zealand; 3 University of Auckland, School of Environment, Auckland, New Zealand; 4 Netherlands Institute for Sea Research (NIOZ-Yerseke), Yerseke, The Netherlands; Université du Québec à Rimouski, Canada

## Abstract

Habitat-modifying organisms that impact other organisms and local functioning are important in determining ecosystem resilience. However, it is often unclear how the outcome of interactions performed by key species varies depending on the spatial and temporal disturbance context which makes the prediction of disturbance-driven regime shifts difficult. We investigated the strength and generality of effects of the filter feeding cockle *Cerastoderma edule* on its ambient intertidal benthic physical and biological environment. By comparing the magnitude of the effect of experimental cockle removal between a non-cohesive and a sheltered cohesive sediment in two different periods of the year, we show that the outcome of cockle interference effects relates to differences in physical disturbance, and to temporal changes in suspended sediment load and ontogenetic changes in organism traits. Interference effects were only present in the cohesive sediments, though the effects varied seasonally. Cockle presence decreased only the density of surface-dwelling species suggesting that interference effects were particularly mediated by bioturbation of the surface sediments. Furthermore, density reductions in the presence of cockles were most pronounced during the season when larvae and juveniles were present, suggesting that these life history stages are most vulnerable to interference competition. We further illustrate that cockles may enhance benthic microalgal biomass, most likely through the reduction of surface-dwelling grazing species, especially in periods with high sediment load and supposedly also high bioturbation rates. Our results emphasize that the physical disturbance of the sediment may obliterate biotic interactions, and that temporal changes in environmental stressors, such as suspended sediments, may affect the outcome of key species interference effects at the local scale. Consequently, natural processes and anthropogenic activities that change bed shear stress and sediment dynamics in coastal soft-sediment systems will affect cockle-mediated influences on ecosystem properties and therefore the resilience of these systems to environmental change.

## Introduction

Coastal soft-sediment habitats such as estuarine tidal flats represent some of our most valued ecosystems and are characterized by a high biomass of invertebrate benthic organisms that sustain coastal foodwebs [1], [2]. However, these productive systems increasingly experience disturbances with the expanding human population and exploitation of coastal areas [3], [4]. For example, changes in sedimentation regimes and water-borne suspended sediment concentrations (SSC) are associated with changes in land-use and engineering activities in the coastal zone, and increased rates of sediment loading adversely affect coastal soft-sediment ecosystem biodiversity and ecological value [5]. Suspended sediments may deposit or remain suspended depending on the physical forces associated with waves and currents, which may vary spatially and temporally and hence affect the balance between deposition and (re)suspension. Nevertheless, both processes influence sediment community composition and functioning. Numerous studies illustrate that terrigenous sediment deposition affects coastal soft-sediment community composition, dynamics, species behavior and ecosystem functioning [6], [7], [8]. Furthermore, suspended sediments decrease the fitness of filter feeding organisms directly by decreasing filtration rate and absorption efficiency that reduces net energy intake [9], and indirectly by increasing turbidity and hence decreasing water column primary production, i.e. food resource availability. Moreover, changes in sediment dynamics that impact the condition and behavior of key species that are involved in maintaining ecosystem resilience, such as habitat-modifying or ecosystem engineering organisms, *sensu* Jones et al. [10], e.g. [11], have the potential to shift the interactions within an ecosystem [12], thereby altering ecosystem stability. This is likely to have far-reaching consequences since the resilience of ecosystems represents an insurance against potentially adverse changes in the delivery of ecosystem goods and services.

In general, experimental field studies indicate strong context-dependency in the strength of ecosystem engineering effects mediated by environmental setting. For example, Norkko et al. [13] illustrate that facilitation strength of a filter feeding bivalve on benthic communities decreased with increased suspended sediment concentration in the water column, while Thrush et al. [14] indicate that the negative effects of a deposit feeding bivalve on juvenile bivalves increased with hydrodynamic stress. In addition, the magnitude of effects may also depend on the local community composition and the strength of specific species interactions. For example, species living in the upper few centimeters of the sediment are particularly affected by sediment erosion [15] and organisms that affect sediment erodibility may therefore particularly affect survival and distribution of juveniles which are unable to burrow deep, thereby compromising natural demographic population processes. The examples above highlight the importance of integrating spatio-temporal dependency in experimental designs to investigate how stressor-mediated changes in behavior of habitat-modifying organisms affect ecosystem properties.

The filter feeding cockle *Cerastoderma edule* (Mollusca: Pelecypoda) lives buried in the top few centimeters of coastal sediments where it can occur in densities >1000 ind.m^−2^ [16], [17]. Field observations indicate a shift towards a less diverse assemblage dominated by deep-living species where *C. edule* is abundant [18]. This species modifies its habitat by altering nutrient processing, depleting the water column of food and larvae, and by changing composition and resuspension of the sediment via vertical and horizontal movements, namely shaking and valve clapping [19]–[21]. Furthermore, mesoscosm experiments reveal that enhanced suspended sediments may affect the outcome of cockle interactions within the ecosystem because suspended sediments stimulate shell shaking and valve clapping [22] with implications for biodiversity and ecosystem functioning (e.g. interference competition and sediment resuspension). Consequently, *C. edule* can be viewed within a network of direct and indirect interactions that support ecosystem functioning and biodiversity which are mediated by bio-physical processes that define intrinsic ecological dynamics.

There is growing evidence that interactions between the impact of stressors and key species-mediated ecological dynamics can initiate threshold responses and increase the risk of a regime shift ([23] and references therein). Consequently, gaining insights in stressor-mediated outcomes of ecosystem engineering interactions will contribute to a better assessment of the stability of functioning in soft-sedimentary habitats that are subject to environmental change, i.e. resilience. This study investigated the strength and generality of the effects of *C. edule* on its intertidal physical and biological environment and contrasted these effects between two different environmental settings. We compared the magnitude of the effect of experimental cockle removal between cohesive and non-cohesive sediments during the recruitment and post-recruitment period of the same year. Comparing the outcome of cockle interference between a strongly physically stressed non-cohesive sediment versus a more stable cohesive sediment should enable interpretation of the importance of environmental physical forcing and differences in community composition in determining cockle effects. Inclusion of temporal variability in the study design allows the assessment of effects on temporally available juveniles, and the importance of temporal changes in suspended sediment concentrations in governing cockle interference effects. We expected that cockle effects on sediment physical and biological properties would be most pronounced in periods when juvenile organisms are present and suspended sediment concentrations are highest, though physical disturbance of the sediment bed resulting from hydrodynamic forces may overwhelm local interference effects.

## Materials and Methods

### Study sites: physical conditions and ambient cockle populations

**Figure 1:**
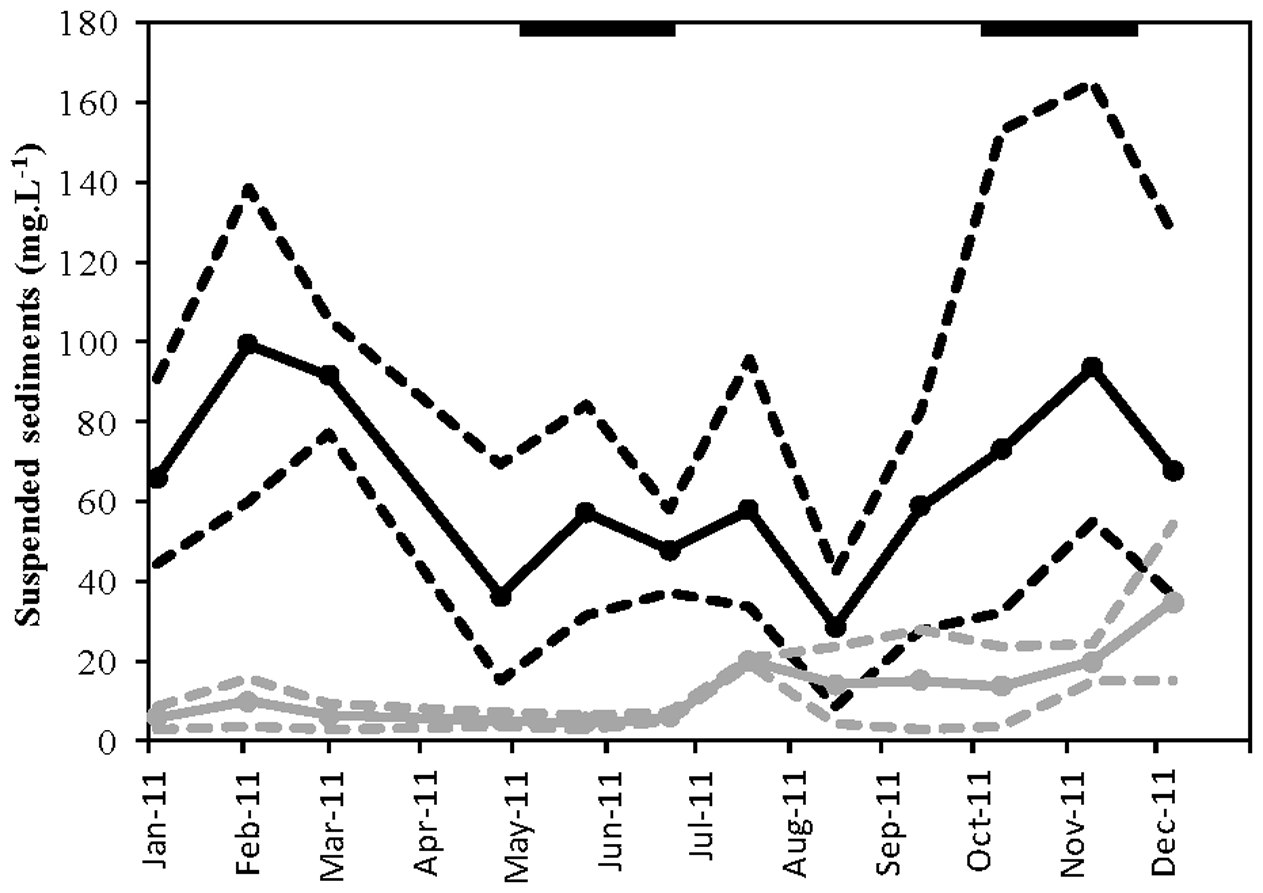
Temporal variation in suspended sediment concentration (SSC) at both study sites in 2011. Black bars indicate the two experimental periods. SSC were retrieved from the public url: www.waterbase.nl. SSC at Paulina (black lines) was calculated as the average value from recordings at Terneuzen, Vlissingen and Hansweert, whereas SSC at Viane (grey lines) was calculated as the average value from recordings at Zijpe and Wissenkerke; see Figure S1 in File S1 for indication of sampling locations. Solid lines indicate average concentrations, dashed lines the minimum and maximum concentration recorded at a sampling location.

**Figure 2:**
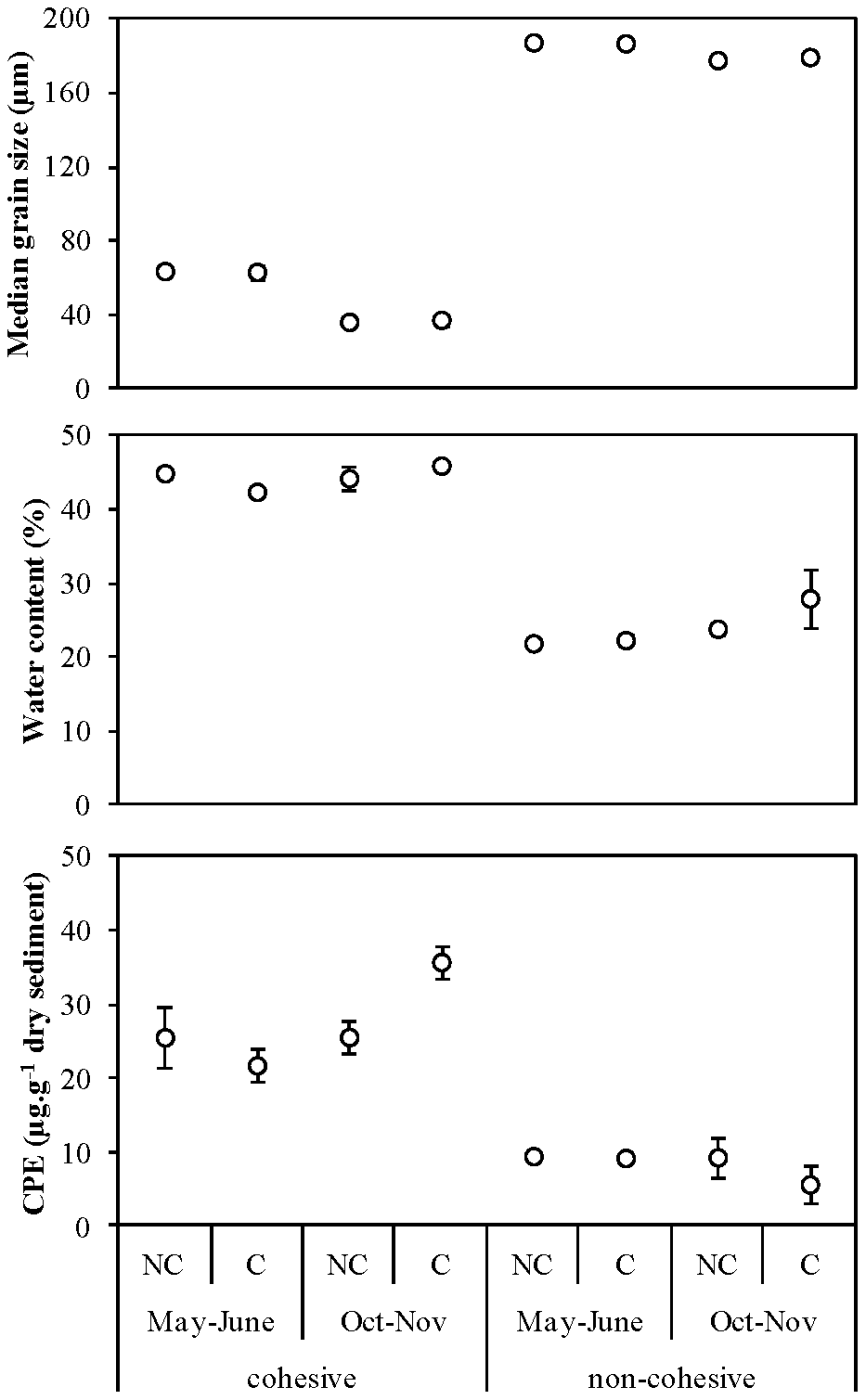
Spatio-temporal effects of cockle treatment on physical sediment properties. Sediment median grain size, water content, and chloroplastic photopigment equivalent (CPE) concentration, shown as mean ± SE for plots without (NC) and with (C) cockles in the two contrasting sediments during both experimental trials, i.e. during recruitment (May–June) and post-recruitment (October–November).

Cockle densities were manipulated in 2011 at two study sites, which are characterized by contrasting physical and biological properties (see results and below). One study site was located in the mid-intertidal zone of the Paulinaschor, in the Westerschelde estuary, whereas the other study site was located in the mid-intertidal zone of the tidal flat near Viane in the Oosterschelde estuary (Figure S1 in File S1). Emersion time at both study sites varied between 50–60%, but the study site at Viane was more exposed to the predominant southwestern winds and experienced higher tidal current velocities (30–40 cm.s^−1^) than the study site at Paulinaschor (20–25 cm.s^−1^). Suspended sediment concentrations (SSC) were higher at Paulinaschor (8.56–165 mg.L^−1^), then at Viane (2.71–54.1 mg.L^−1^) over the course of the experiment. In general, SSC was about twice as high in autumn and winter than in spring and summer at both study sites (Figure 1). Sediment at Paulinaschor was cohesive and consisted predominantly of silt (median grain size = 49.4 µm), whereas sediment at Viane was non-cohesive consisting predominantly of fine and medium sand (median grain size = 184.9 µm). In addition, the non-cohesive sediment contained less pore water and lower organic matter derived from primary production, i.e. chloroplastic photopigment equivalents (CPE) (Figure 2). Study sites are further referred to as cohesive site (Paulinaschor) and non-cohesive site (Viane).

The adult cockle population in 2011 consisted of two cohorts (recruited in 2009 and 2010) at both sites. Average adult density was 16.9±1.6 SE ind.0.25 m^−2^ at the non-cohesive site and 18.0±7.5 ind.0.25 m^−2^ at the cohesive site.

### Experimental design and sample processing

Four paired 0.5 m×0.5 m (i.e. 0.25 m^2^) experimental plots were selected along a 25 m transect which was randomly placed parallel to the tide line in the mid intertidal zone of both study sites. Distances between pairs of plots was 5 m. Cockle densities were manipulated by incubating each plot for 10 days under a nylon 1 mm mesh-sized net, that was placed flush with the sediment surface, using metal frames. This technique chased cockles to the sediment surface so that they could easily and effectively be removed. During the same low tide, 17 adult alive cockles (i.e. average ambient adult density) that were collected at the study site were added into one randomly assigned plot per pair, yielding one plot with cockles (C) and one plot without cockles (NC) per pair. Cockles were added using a 0.5×0.5 m frame with 25 10×10 cm quadrats, ensuring a homogenous distribution of the study organisms at the start of the experiment. We performed the experiment in two different periods of the year; i.e. during recruitment (May–June, frame deployment on April 18^th^ and 19^th^) and post-recruitment (October–November, frame deployment on September 22^nd^ and 23^th^) at both study sites [24], [25]. The average size of added cockles was 28.9±0.1 SE mm at the non-cohesive site for the trial during the recruitment period, and 28.0±0.1 SE mm for the post-recruitment trial. At the cohesive site, the size of added cockles was 28.6±0.1 SE mm for the trial during recruitment, and 29.4±0.1 SE mm for the post-recruitment trial.

Two days after removal of the frames, we collected samples in the outer rim of each NC plot and from the adjacent ambient unmanipulated sediment to assess potential artifacts on sediment physical and biological properties associated with deployment of the frames and nets. Deployment of frames did not significantly influenced sediment particle size, water content, redox potential discontinuity layer depth, and macrofauna community composition (except adult cockles) during both study periods at both sites, though CPE was slightly lower in the plots as compared to the ambient sediment at the start of the post-recruitment trial, i.e. respectively −6.9 µg.g^−1^ and −1.9 µg.g^−1^ at the cohesive and the non-cohesive site (Table S1 in File S1).

Cockle effects on sediment physical and biological properties, and the number of adult cockles present in the plots, were determined after six weeks. The number of adult cockles was assessed by sieving the inner 30 cm diameter of each plot. Adult cockle densities in the inner 30 cm diameter of the C plots after six weeks was, on average, respectively 4.8 and 5.0 during the recruitment trial and the post-recruitment trial in the non-cohesive sediments, and respectively 3.8 and 4.0 in the cohesive sediments during the recruitment and post-recruitment trial. Cockle densities thus equaled 13–17 individuals per C plot across all treatments and trials, while only one adult cockle was found in all NC plots. Cockle effects on benthic communities were determined from three 10 cm (i.d.) perspex corers that were randomly collected to a depth of 10 cm in the inner 30 cm diameter of each C and NC plot. These samples were pooled and fixed on a neutralized 8% formaldehyde-tapwater solution. Subsequently, samples were sieved over a 1 mm mesh in the laboratory, and the organism retained on the sieve were counted and identified to the lowest possible taxonomic level. Similarly, we collected three 3.2 cm (i.d.) perspex corers to a depth of 5 cm from the inner 30 cm of each plot in order to quantify sediment properties. From each corer we stored the upper 0.5 cm of sediment on dry ice and at −20°C (for sediment composition and water content, two samples) and −80°C (for CPE concentrations, one sample) upon arrival in the laboratory awaiting further processing. Samples were weighed wet, lyophilized in the dark and weighed again to yield absolute water content (%). Sediment composition was determined from the dry sediment using Malvern laser diffraction and CPE, i.e. sum of chlorophyll a and its degradation products (phaeopigments), was determined following Jeffrey et al. [26] using HPLC of the supernatant that was extracted from the freeze-dried sediment by adding 10 ml 90% acetone.

### Data analysis

Two-way Analysis of Similarities [27] was used to compare community composition between study sites and experimental trials, using the samples from the ambient sediment that were collected two days after removal of the frames.

We compared the magnitude of the effect of cockle presence (C) versus experimental cockle removal (NC) in two contrasting sediment types. Given the general higher variability in non-cohesive sediments and the difference in dominant species presence between both sediment types, we analyzed the effects of cockle interference using a two-way factorial generalized linear model for each sediment type separately. This model included the fixed factors Cockle (present, removed) and Timing (recruitment, post-recruitment period), as timing of the experiment was deliberately chosen to represent seasonal changes in SSC and presence of juveniles which may affect the outcome of cockle interference on sediment properties. Gamma error structures, using a log-link function were used for models of total density and species density, while normal error structures were used for general community attributes (species richness, Pielou's evenness, and Simpson's index of diversity) and physical sediment properties. Densities are expressed as individuals per 0.024 m^−2^, i.e. the total surface of the three pooled corers. We removed typical meio-, hyper- and epifaunal taxa from the biotic dataset since these organisms were not sampled qualitatively. Log_e_ transformation was applied to meet the assumption of within group variance homogeneity (Levene test), if needed. Alternatively, if Log_e_ transformation was not successful in producing homogeneity of variance, we conducted two-way factorial permutational analysis of variance after data similarities were calculated using Euclidean distances, and checked for multivariate dispersion [28].We assessed cockle effects on the density of the five most dominant species from each site, which comprised 91 and 71% of the total density at the cohesive and non-cohesive site, respectively. Since we expected that surface-dwelling organisms that feed on the surface sediment and that have rather limited dispersal capacities during their adult life stage would be particularly influenced by cockles, we additionally analyzed cockle effects on the diversity and density of this pool of organisms separately. These surface deposit feeders that have limited dispersal capacities as compared to the highly mobile amphipods are further referred to as LDSDF.

## Results

### Species assemblage of non-cohesive and cohesive sediments

Benthic community composition differed significantly among study sites and over time (ANOSIM, site R = 0.995, p<0.001; time R = 0.289, p = 0.003). Overall, Polychaeta (4 species), and Crustacea (3 species) comprised 88% of the individuals present at the non-cohesive study site, while Polychaeta (8 species) and Oligochaeta spp. were the most dominant taxa at the cohesive study site comprising, respectively, 88 and 7% of the total number of individuals. At the cohesive site, LDSDF comprised 86% of the total community, while only 59% of the organisms were LDSDF at the non-cohesive site.

**Figure 3:**
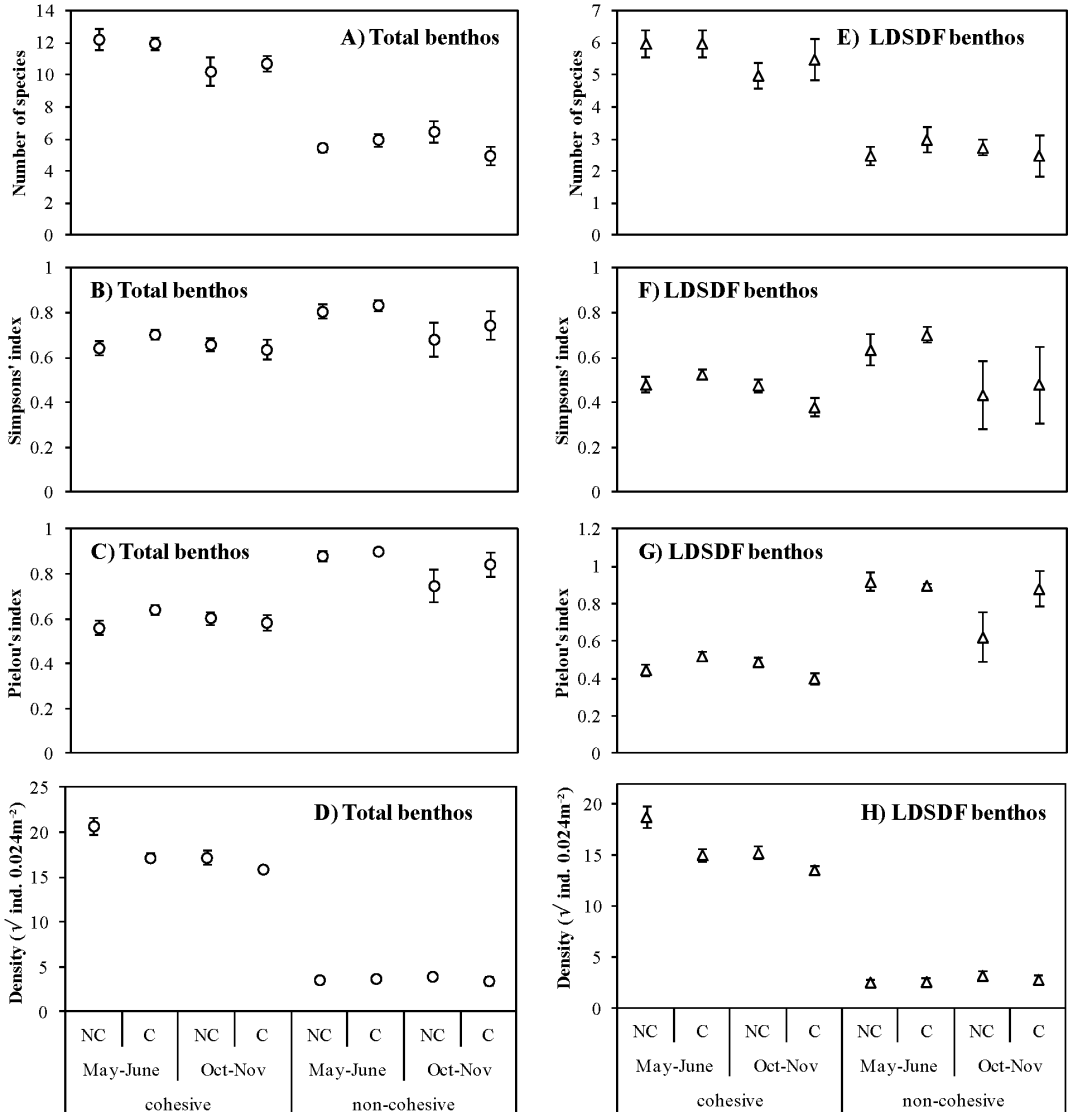
Spatio-temporal effects of cockle treatment on biological sediment properties. Species richness, diversity, evenness and total density for the total community (A–D) and the less-mobile surface deposit feeders (LDSDF, E–H); shown as mean ± SE for plots without (NC) and with (C) cockles in the two contrasting sediments during both experimental trials, i.e. during recruitment (May–June) and post-recruitment (October–November). Note that presented densities are square root transformed.

During both trials, a higher total density and species richness were present in the cohesive sediments, but total community diversity and evenness was higher in the non-cohesive sediments (Figure 3, a–d). Similarly, LDSDF species richness and total density were higher in the non-cohesive sediments, while LDSDF evenness was higher during both trials, and LDSDF diversity was higher during in May–June, in the non-cohesive sediments (Figure 3, e–f). The five most dominant species at the cohesive site were the LDSDF *Aphelochaeta marioni*, *Pygospio elegans* and juvenile recruits of *Macoma balthica*, and the subsurface deposit feeding species *Heteromastus filiformis* and Oligochaeta spp. In contrast, the LDSDF species *Scoloplos armiger* and *Pygospio elegans*, the mobile species *Hydrobia ulvae* and *Urothoe poseidonis*, and the filter feeding recruits of *C. edule* were the most dominant species at the non-cohesive site.

### Cockle effects in cohesive sediments

**Figure 4:**
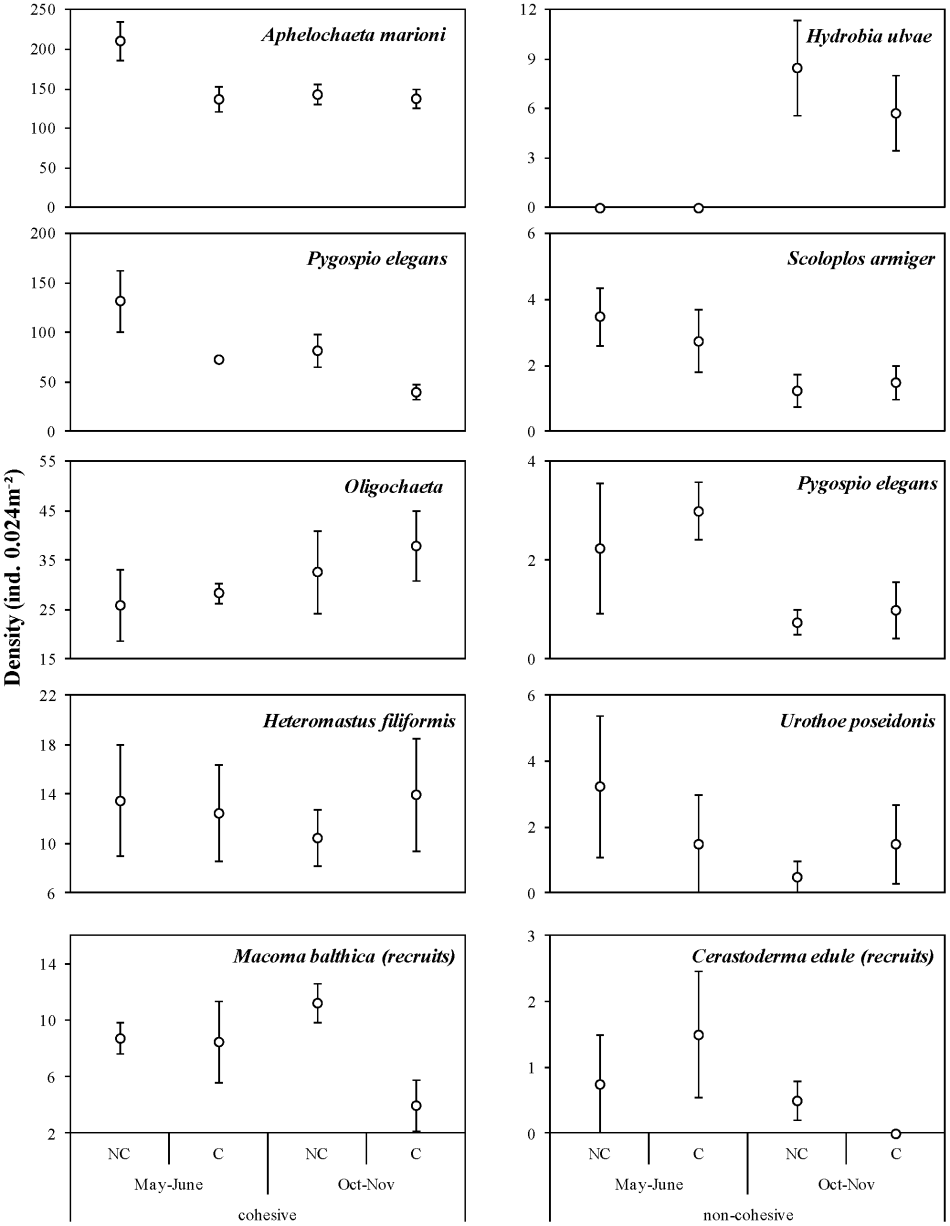
Temporal effects of cockle treatment on the density of the five most dominant species per study site. Densities are shown as mean ± SE for plots without (NC) and with (C) cockles during both experimental trials, i.e. during recruitment (May–June) and post-recruitment (October–November) in the cohesive sediments (left panel) and in the non-cohesive sediments (right panel).

Median grain size decreased during the post-recruitment trial, independent of cockle treatment (Figure 2). Cockles significantly affected physical and biological sediment properties, though the effects often varied temporally. Cockle presence significantly enhanced CPE during the post-recruitment trial in October–November (Figure 2) (Table S2 in File S1). Furthermore, total macrobenthos and LDSDF density was reduced when cockles were present, though this decrease was more pronounced during the recruitment trial in May–June (Figure 3). Cockle presence significantly decreased the density of *A. marioni*, *P. elegans* and juveniles of *M. balthica* during at least one of the trials (Table S2 in File S1). Density of *A. marioni* was reduced by 35% during the recruitment trial, while density of *M. balthica* recruits was reduced by 65% during the post-recruitment trial (Figure 4). The density of *P. elegans* was, on average, reduced by 45% in May–June and by 51% in October–November. No detectable effects were apparent for Oligochaeta spp. and *H. filiformis*. Diversity and evenness of the LDSDF community was lowest in the C plots at the end of the post-recruitment trial (Figure 3).

### Cockle effects in non-cohesive sediments

None of the measured physical and biological sediment properties was significantly influenced by the cockle treatment in the non-cohesive sediments (Table S3 in File S1). However, some sediment properties revealed significant temporal variation, independent of cockle presence. Similar to the cohesive sediments, median grain size decreased during the post-recruitment trial (Figure 2). In general, the density of the most dominant species was lower during the post-recruitment trial in October–November, except for *Hydrobia ulvae* (Figure 4). The latter species was absent in May–June, but was the most dominant member of the community in October–November. However, these changes in species density did not significantly affected overall and LDSDF community density, diversity and evenness.

## Discussion

We have examined the generality of the interaction between a filter feeding infaunal bivalve, *C. edule*, and its ambient biotic and physical environment. By manipulating cockle densities in two contrasting sediment types (i.e. a sheltered cohesive tidal flat *vs*. a physically stressed non-cohesive tidal flat) in two different periods (i.e. during recruitment and post-recruitment phase) we were able to relate the significance of cockle effects to physical disturbance, and to temporal changes in the presence of species life stages and likely changes in cockle behavior.

Firstly, we demonstrate the temporal dependence of the strength and outcome of cockle effects on physical and biological sediment properties. We suggest two aspects as being governing drivers of the observed temporal effect: (1) species-specific life-stage dependent vulnerability to cockle interference, and (2) changing cockle behavior associated with increasing SSC. We found reduced densities of the dominant surface-dwelling species when cockles were present in the cohesive sediments. Lower densities may have resulted from both direct interference competition and active post-settlement dispersal [29], [30]. The absence of an effect of cockle removal on the subsurface-dwelling Oligochaeta spp. and *H. filiformis* suggests that cockle effects on benthic communities in cohesive sediments are mainly mediated by interference effects of cockle bioturbation in the surface sediment layer. Furthermore, densities of the LDSDF *A. marioni* and *P. elegans* were numerically less reduced in the C plots during the post-recruitment trial, than during the recruitment trial which suggests that larvae and recently recruited juveniles of these species are more vulnerable to cockle interference as compared to 4–5 months old juveniles. This is particularly clear for *A. marioni* which was reduced by 35% during the recruitment trial, while the population density of this species was not affected by cockle presence or removal during the post-recruitment trial. This species has a direct larval benthic development (www.marlin.ac.uk) and is thus potentially highly vulnerable to disturbance of the sediment during its larval stage. Average suspended sediment concentrations increased at the cohesive study site from 47.8–57.3 mg.L^−1^ in May–June to 73.3–93.9 mg.L^−1^ in October–November (i.e. post-recruitment), with a recorded maximum concentration of 165.0 mg.L^−1^ in November. This is a yearly phenomenon [31], [32] and is most likely associated with enhanced rainfall and associated soil erosion in the Sheldt catchment area in this period of the year [32]. Ciutat et al. [22] empirically demonstrated that *C. edule* increases its frequency of valve clapping with increasing SSC in order to eject excess sediments from its mantle cavity. This behavior generally increases linearly with the increase in SSC until ∼300 mg.L^−1^ [22]. Populations of *P. elegans* and juveniles of *M. balthica* were significantly reduced when cockles were present in the period with enhanced SSC and thus supposedly also higher cockle bioturbation rates. Both species rely substantially on microalgal carbon for their diet at Paulinaschor [18], [33]. Hence, the observed higher CPE concentration in the C plots during the post-recruitment trial may therefore result from enhanced cockle bioturbation-mediated decreases in density, and thus grazing pressure, of *P. elegans* and *M. balthica*. The two numerically dominant species in the community, *A. marioni* and *P. elegans*, thus exhibited a different response to cockle presence during the post-recruitment trial in autumn. Because no species were outcompeted by cockles (i.e. total species number remained constant in C and NC plots) but, of the two species, only *P. elegans* was inhibited by cockle presence during the post-recruitment trial, evenness and diversity decreased during this trial.

Secondly, our study demonstrates the potential for physical disturbance resulting from hydrodynamic forces to overwhelm the effects of cockles on physical and biological sediment properties. Above a threshold, physical factors are considered relatively more important than biotic interactions in mediating community organization (e.g. [34], [35]). Though *C. edule* clearly has the potential to affect its physical and biological environment, we conclude that the physical disturbance of the sediment bed associated with hydrodynamic forces at the non-cohesive study site, which has consequences for organism dispersal and species population demographics [15], [36], likely obliterated the ecosystem engineering of *C. edule* on the surface sediment layers. In general, differences in benthic biological community attributes and physical sediment properties were greater between the two contrasting sediment types during both experimental trials, than between when cockles were present or removed (Figures 2, 3). This illustrates that physical disturbance resulting from wave action and tidal currents is a governing process of estuarine benthic diversity and sediment properties (e.g. [18], [37]). The stability of bed sediment depends on the balance between hydrodynamic forces that cause erosion and the forces within the sediment that resist it, with consolidated sediment that contain fine particles (e.g. mud, i.e. particles <63 µm) typically having a lower propensity to be eroded then unconsolidated, non-cohesive sediments (Grabowski et al. [38], and references therein).

In conclusion, our results emphasize that physical disturbance of the sediment bed due to hydrodynamic forcing, can constrain the outcome of biotic interactions at the local scale, and that temporal changes in environmental stressors, e.g. suspended sediment concentrations, may affect the outcome of key species interference effects, e.g. through changes in key species behavior and life-stage specific vulnerability to interference from bioturbation. As a consequence, the obtained results suggest that inconsistencies among previous studies that examined the effect of cockle loss or manipulated cockle densities on benthic communities (see e.g. [39]–[41]) may result from differences in the strength and outcome of interference effects depending on the spatial and temporal context. Experiments conducted at multiple sites and times should therefore be considered preferable in order to generalize results of how key species affect ecosystem properties [42], therewith enhancing our understanding of ecosystem resilience in the context of spatio-temporal variable stressors. Consequently, the obtained insights into the context-dependent effects of cockles on biotic and abiotic sediment properties in this study are relevant to assess shallow coastal ecosystem stability, in particular for those systems that are subjected to changing sediment dynamics. This study illustrates that processes that alter bed shear stress and sediment load in coastal cohesive soft-sediment systems (e.g. changes in hydro-morphology, storm frequency and intensity, terrigeneous sediment run-off, etc.) will affect cockle-mediated influences on ecosystem properties, impairing the resilience of ecosystem functioning.

## Supporting Information

File S1 Figure S1, Location of the study sites (transect lines in white) in the mid intertidal zone at Paulinaschor and Viane, and locations for monthly samplings of suspended sediment concentrations (red circles) in the subtidal channel. Table S1, Three-way factorial permutational anova results of the effect of plot incubation (Factor: Location, i.e. in or out the plot) on physical (water content, chloroplastic photopigment equivalent concentrations, median grain size) and biological (community structure) sediment properties at the two sites (Factor: Site) for the two experimental trials (Factor: Trial). Boldfaced p-values indicate significant effect at p<0.05. Table S2, Two-way factorial analysis of variance results (p-values) of the effect of cockle removal or presence (Factor: Treatment) on physical and biological sediment properties during the two experimental trials (Factor: Trial) at the cohesive study site. Boldfaced p-values indicate significant effect at p<0.05. Table S3, Two-way factorial analysis of variance results (p-values) of the effect of cockle removal or presence (Factor: Treatment) on physical and biological sediment properties during the two experimental trials (Factor: Trial) at the non-cohesive study site. ^p^results from permutational analysis of variance. Boldfaced p-values indicate significant effect at p<0.05.
(DOCX)
