# Supplementary material for: Conditional Responses of Benthic Communities to Interference from an Intertidal Bivalve
Source: PLoS One. 2013 Jun 18;8(6):e65861. doi: 10.1371/journal.pone.0065861 (PMC3688828; doi:10.1371/journal.pone.0065861)
Supplement: File S1 — Figure S1, Location of the study sites (transect lines in white) in the mid intertidal zone at Paulinaschor and Viane, and locations for monthly samplings of suspended sediment concentrations (red circles) in the subtidal channel. Table S1, Three-way factorial permutational anova results of the effect of plot incubation (Factor: Location, i.e. in or out the plot) on physical (water content, chloroplastic photopigment equivalent concentrations, median grain size) and biological (community structure) sediment properties at the two sites (Factor: Site) for the two experimental trials (Factor: Trial). Boldfaced p-values indicate significant effect at p<0.05. Table S2, Two-way factorial analysis of variance results (p-values) of the effect of cockle removal or presence (Factor: Treatment) on physical and biological sediment properties during the two experimental trials (Factor: Trial) at the cohesive study site. Boldfaced p-values indicate significant effect at p<0.05. Table S3, Two-way factorial analysis of variance results (p-values) of the effect of cockle removal or presence (Factor: Treatment) on physical and biological sediment properties during the two experimental trials (Factor: Trial) at the non-cohesive study site. presults from permutational analysis of variance. Boldfaced p-values indicate significant effect at p<0.05. (DOCX) [file pone.0065861.s001.docx]

**File S1**

**Supporting information to:**

Conditional responses of benthic communities to interference from an intertidal bivalve

Van Colen C., Thrush S.F., Vincx M., Ysebaert T.

**Figure S1**

**Table S1**

| Factor | df | SS | MS | Pseudo-F | P(perm) | P(MC) |
| --- | --- | --- | --- | --- | --- | --- |
|  |  |  |  |  |  |  |
| ***RPDZ*** |  |  |  |  |  |  |
| Site (Si) | 1 | 29.3570 | 29.3570 | 19.3600 | 0.0002 | **0.0004** |
| Trial (Tr) | 1 | 19.4530 | 19.4530 | 12.8290 | 0.0005 | **0.0009** |
| Location (Lo) | 1 | 1.3958 | 1.3958 | 0.9205 | 0.3721 | 0.3481 |
| sixtr | 1 | 0.6375 | 0.6375 | 0.4204 | 0.5451 | 0.5162 |
| sixlo | 1 | 0.4713 | 0.4713 | 0.3108 | 0.6110 | 0.5775 |
| trxlo | 1 | 3.0525 | 3.0525 | 2.0130 | 0.1740 | 0.1704 |
| sixtrxlo | 1 | 0.8504 | 0.8504 | 0.5608 | 0.4866 | 0.4634 |
| Res | 24 | 36.3930 | 1.5164 |  |  |  |
| Total | 31 | 91.6110 |  |  |  |  |
|  |  |  |  |  |  |  |
| ***Median grain size*** |  |  |  |  |  |  |
| Site (Si) | 1 | 146950.0000 | 146950.0000 | 3265.6000 | 0.0001 | **0.0001** |
| Trial (Tr) | 1 | 1618.2000 | 1618.2000 | 35.9600 | 0.0001 | **0.0001** |
| Location (Lo) | 1 | 41.6740 | 41.6740 | 0.9261 | 0.3614 | 0.3477 |
| sixtr | 1 | 87.3640 | 87.3640 | 1.9415 | 0.1822 | 0.1771 |
| sixlo | 1 | 22.4450 | 22.4450 | 0.4988 | 0.5006 | 0.4888 |
| trxlo | 1 | 0.0228 | 0.0228 | 0.0005 | 0.9836 | 0.9813 |
| sixtrxlo | 1 | 2.2834 | 2.2834 | 0.0507 | 0.8391 | 0.8242 |
| Res | 24 | 1080.0000 | 44.9990 |  |  |  |
| Total | 31 | 149800.0000 |  |  |  |  |
|  |  |  |  |  |  |  |
| ***Water content*** |  |  |  |  |  |  |
| Site (Si) | 1 | 0.1923 | 0.1923 | 216.3700 | 0.0001 | **0.0001** |
| Trial (Tr) | 1 | 0.0055 | 0.0055 | 6.2167 | 0.0197 | **0.0193** |
| Location (Lo) | 1 | 0.0008 | 0.0008 | 0.8963 | 0.3573 | 0.3566 |
| sixtr | 1 | 0.0039 | 0.0039 | 4.3692 | 0.0466 | **0.0460** |
| sixlo | 1 | 0.0005 | 0.0005 | 0.5970 | 0.4452 | 0.4462 |
| trxlo | 1 | 0.0004 | 0.0004 | 0.4202 | 0.5245 | 0.5260 |
| sixtrxlo | 1 | 0.0001 | 0.0001 | 0.0751 | 0.7882 | 0.7787 |
| Res | 24 | 0.0213 | 0.0009 |  |  |  |
| Total | 31 | 0.2248 |  |  |  |  |
|  |  |  |  |  |  |  |
| ***CPE*** |  |  |  |  |  |  |
| Site (Si) | 1 | 423.3100 | 423.3100 | 42.1350 | 0.0001 | **0.0001** |
| Trial (Tr) | 1 | 636.4400 | 636.4400 | 63.3500 | 0.0001 | **0.0001** |
| Location (Lo) | 1 | 19.4700 | 19.4700 | 1.9381 | 0.1740 | 0.1719 |
| sixtr | 1 | 46.7740 | 46.7740 | 4.6558 | 0.0362 | **0.0413** |
| sixlo | 1 | 30.4360 | 30.4360 | 3.0296 | 0.0936 | 0.0920 |
| trxlo | 1 | 64.0940 | 64.0940 | 6.3798 | 0.0125 | **0.0170** |
| sixtrxlo | 1 | 2.4291 | 2.4291 | 0.2418 | 0.6243 | 0.6159 |
| Res | 24 | 241.1100 | 10.0460 |  |  |  |
| Total | 31 | 1464.1000 |  |  |  |  |
|  |  |  |  |  |  |  |
| ***Community structure*** |  |  |  |  |  |  |
| Site (Si) | 1 | 42398.0000 | 42398.0000 | 49.2580 | 0.0001 | **0.0001** |
| Trial (Tr) | 1 | 3507.6000 | 3507.6000 | 4.0752 | 0.0003 | **0.0021** |
| Location (Lo) | 1 | 543.9300 | 543.9300 | 0.6320 | 0.7245 | 0.6895 |
| sixtr | 1 | 2288.1000 | 2288.1000 | 2.6583 | 0.0133 | **0.0249** |
| sixlo | 1 | 140.5300 | 140.5300 | 0.1633 | 0.9727 | 0.9679 |
| trxlo | 1 | 349.7100 | 349.7100 | 0.4063 | 0.8753 | 0.8437 |
| sixtrxlo | 1 | 596.3600 | 596.3600 | 0.6929 | 0.6653 | 0.6260 |
| Res | 24 | 20657.0000 | 860.7200 |  |  |  |
| Total | 31 | 70481.0000 |  |  |  |  |

**Table S2**

|  | Treatment | Trial | Treatment x Trial |
| --- | --- | --- | --- |
| Water content | 0.722 | 0.214 | 0.066 |
| CPE | 0.461 | 0.058 | **0.016** |
| Median grain size | 0.893 | **<0.001** | 0.825 |
| Number of species - total benthos | 0.843 | **0.022** | 0.554 |
| Simpsons' index - total benthos | 0.584 | 0.448 | 0.233 |
| Pielou's index - total benthos | 0.313 | 0.824 | 0.100 |
| Abundance - total benthos | **<0.001** | **<0.001** | 0.077 |
| Number of species - LDSDF | 0.611 | 0.143 | 0.611 |
| Simpsons' index - LDSDF | 0.423 | **0.040** | **0.048** |
| Pielou's index - LDSDF | 0.798 | 0.181 | **0.012** |
| Abundance - LDSDF | **<0.001** | **<0.001** | 0.144 |
| *Aphelochaeta marioni* | **0.010** | **0.035** | **0.030** |
| *Pygospio elegans* | **<0.001** | **0.001** | 0.707 |
| *Oligochaeta* | 0.502 | 0.148 | 0.874 |
| *Hetermastus filiformis* | 0.691 | 0.795 | 0.492 |
| *Macoma balthica (recruits)* | **0.049** | 0.376 | 0.063 |

**Table S3**

|  | Treatment | Trial | Treatment x Trial |
| --- | --- | --- | --- |
| Water content^p^ | 0.331 | 0.120 | 0.436 |
| CPE^p^ | 0.320 | 0.341 | 0.385 |
| Median grain size^p^ | 0.784 | **0.001** | 0.595 |
| Number of species - total benthos | 0.529 | 0.751 | 0.132 |
| Simpsons' index - total benthos^p^ | 0.395 | 0.068 | 0.750 |
| Pielou's index - total benthos^p^ | 0.239 | 0.073 | 0.439 |
| Abundance - total benthos | 0.585 | 0.830 | 0.390 |
| Number of species - LDSDF | 0.340 | 0.746 | 0.746 |
| Simpsons' index - LDSDF | 0.640 | 0.108 | 0.938 |
| Pielou's index - LDSDF^p^ | 0.203 | 0.098 | 0.138 |
| Abundance - LDSDF^p^ | 0.669 | 0.156 | 0.559 |
| *Hydrobia ulvae* | 0.438 | **0.000** | 0.438 |
| *Scoloplos armiger* | 0.861 | **0.013** | 0.514 |
| *Pygospio elegans* | 0.469 | **0.005** | 0.875 |
| *Urothoe poseidonis* | 0.870 | 0.347 | 0.347 |
| *Cerastoderma edule (recruits)* | 0.922 | **0.031** | 0.124 |
